# Reflections on Identity in Memoirs of Writers With Aphasia: Lessons Learned on the Path Toward Recovery

**DOI:** 10.1093/geroni/igab046.413

**Published:** 2021-12-17

**Authors:** Hanna Ulatowska, Gloria Olness

**Affiliations:** 1 University of Texas at Dallas, Dallas, Texas, United States; 2 University of North Texas, Denton, Texas, United States

## Abstract

Personal stories provide insight into the experience of illness as it intersects with one’s identity. Prior studies by the first author examined identity as manifested in personal accounts of U.S. World War II veterans with and without dementia. The current study examines identity as revealed through written memoirs of middle-aged and older adults who have aphasia, from a cross-section of North American, European, and Australian cultures. The abrupt onset of stroke and associated aphasia, and the subsequent path toward re-engagement in life with an often-chronic communicative impairment, provide a unique window into the nature and evolution of the identity of the writer. The written modality offers an opportunity for reflective formulation that is not afforded to the memoir-writers in their verbal expression. Nineteen memoirs and biographical accounts of individuals with aphasia from a range of primarily individualistic cultures were examined for content reflective of the identity of the author, focused on post-stroke phases of restitution and quest. Primary authors were people with aphasia or rarely their close family member. Some were professional editors, poets or authors. Gender and life backgrounds were varietal. Manifestations of personal identity, its reinforcement, and its evolution were evidenced in: the provision of lessons learned from living with aphasia; content of letters exchanged with friends; engagement with family in life and recovery; fictional and poetic expression; spiritual insight; renewed or altered occupational pursuits; and comments on facing one’s mortality. Findings hold implications for the cross-cultural practice of narrative medicine with the older adult population.

